# Impact of estrogen receptor expression level on response to neoadjuvant chemotherapy and prognosis in HER2-negative breast cancers

**DOI:** 10.1186/s12885-023-11368-2

**Published:** 2023-09-08

**Authors:** Hai-long Chen, Feng-bo Huang, Qiang Chen, Yong-chuan Deng

**Affiliations:** 1 Department of Breast Surgery, the Second Affiliated Hospital of Zhejiang, University School of Medicine, Hangzhou, Zhejiang Province China; 2 Department of Pathology, the Second Affiliated Hospital of Zhejiang University School of Medicine, Hangzhou, Zhejiang Province China

**Keywords:** Breast cancer, Estrogen receptor, Neoadjuvant chemotherapy, Pathologic complete response, Prognosis

## Abstract

**Background:**

Breast cancers with 1–10% cell staining for estrogen receptor (ER) present particular clinical features. The clinical data of estrogen receptor expression level and treatment effect are limited, particularly regarding chemotherapy benefit. We evaluated the pathologic response to neoadjuvant chemotherapy (NAC) in ER low positive tumors (ER staining 1-10%) and compared it with ER > 10% positive tumors (ER staining > 10%) and ER-negative tumors. We further explored the differences in recurrence and survival with respect to the ER expression level.

**Method:**

Patients with stages II and III HER2-negative primary breast cancer who received neoadjuvant chemotherapy followed by definitive surgery were categorized according to their ER percentages into three groups: ER-negative, ER low positive, and ER > 10% positive. Logistic regression models were used to assess the association between each variable and pathologic complete response (pCR). Kaplan‒Meier analysis was used to estimate survival outcomes. Cox models were used to adjust for patient and tumor characteristics.

**Results:**

A total of 241 patients were analyzed. Of all patients included, 22 (9.1%) had ER low positive tumors, 159 (66.0%) had ER > 10% positive tumors, and 60 (24.9%) were ER-negative. Low ER positivity was significantly associated with a higher pCR rate than ER > 10% positivity (OR, 0.249; 95% CI, 0.067–0.923; P = 0.038). After a median follow-up time of 32 months, the disease-free survival (DFS) and overall survival (OS) of the patients with ER low positive tumors were significantly worse than those of the patients with ER > 10% positive tumors but similar to those with ER-negative tumors. After adjustment for covariates, ER low positive tumors were significantly associated with worse DFS than ER > 10% positive tumors.

**Conclusion:**

Our results indicated that ER low positive breast cancer presents a better response to neoadjuvant chemotherapy and significantly worse prognosis for patients than those with ER > 10% positive tumors, but similar to the ER-negative group. These data support that this category of patients behaves clinically like patients with ER-negative breast cancer and should be treated differently from patients with ER > 10% positive tumors. Further prospective study is needed.

**Supplementary Information:**

The online version contains supplementary material available at 10.1186/s12885-023-11368-2.

## Introduction

Estrogen receptor (ER) expression, which is evaluated by immunohistochemistry (IHC), is a prognostic and predictive factor in breast cancer [1, 2]. Approximately 70% of breast cancers are ER-positive, and ER-positive breast cancers manifest higher endocrine therapy sensitivity but less chemotherapy responsiveness than ER-negative breast cancers. In the past, the cutoff percentage value of positively stained cells to define tumors as ER-positive remained controversial, and a large number of investigators traditionally utilized a cutoff of > 10% for determining patient eligibility for endocrine treatment [3–7]. However, a wide range of cutoff values (1-20%) in terms of the percentage of stained cells were used [8–10]. In 2010, the guidelines of the American Society of Clinical Oncology and the College of American Pathologists (ASCO/CAP) defined ER-positive tumors as those with ≥ 1% of tumor cell nuclei exhibiting immunoreactivity because of the substantial impact of endocrine therapies on mortality reduction in these patients [11]. However, the 1% cutoff is not supported by strict evidence, and some studies have revealed that tamoxifen was ineffective with low ER expression [12, 13]. Data from prospectively conducted clinical trials to validate the optimal threshold are still lacking.

Although cancers with 1–10% cell staining for ER are uncommon (ranging from 3 to 9% among all patients) [12, 14], they present particular clinical challenges. The ASCO/CAP recommended that these cancers should be reported using a new reporting category, ER low positive [15]. It was demonstrated that patients with low ER expression have similar clinical and pathologic characteristics to patients with ER-negative tumors [12, 16–19]. A few studies have shown a correlation between the benefit of hormonal therapy and the degree of ER expression [20–22], indicating that more attention should be given to the degree of ER positivity. However, the clinical data of estrogen receptor expression level and treatment effect were limited, particularly regarding chemotherapy benefit. Moreover, most clinical trials only divided tumors into ER-positive and ER-negative groups, without focusing on the degree of ER positivity. In this study, we evaluated the pathologic response to neoadjuvant chemotherapy (NAC) in ER low positive tumors (ER staining 1-10%) and compared it with ER > 10% positive tumors (ER staining > 10%) and ER-negative tumors. We further explored the differences in recurrence and survival with respect to the degree of ER expression.

## Patients and methods

### Patients

We retrospectively analyzed breast cancer tumors in patients treated with NAC from January 2016 to December 2020 at the Second Affiliated Hospital, Zhejiang University (Hangzhou, China). Patients were included if they had histologically confirmed breast cancer (stages II-III) and were treated with NAC before definitive breast and axillary surgery. The following exclusion criteria were applied for patient selection: (1) lack of ER IHC results, (2) cases that were previously treated with chemotherapy, radiation therapy or targeted therapy, (3) cases with other malignancies or bilateral breast cancer and (4) HER2-positive cases. Demographic, clinicopathologic, and treatment data were retrospectively collected from medical charts.

This study was approved by the Ethics Committee of the Second Affiliated Hospital of Zhejiang University School of Medicine (Hangzhou, China; approval no. 2022-0094). The need for informed consent was waived by the Ethics Committee because the study was an observational, retrospective study, and the patients’ identification information was removed.

### Immunohistochemistry assessment

Tumor core biopsies were performed before NAC therapy commenced. ER, PgR, Ki-67 and HER2 were assessed by IHC. IHC staining for ER (Confirm anti-ER, rabbit monoclonal primary antibody; clone SP1, Ventana Medical Systems), PR (Confirm anti-PR, rabbit monoclonal primary antibody; clone 1E2; Ventana Medical Systems), ki-67 (mouse monoclonal primary antibody; clone MIB1; Origene), and HER2 (anti-HER2/neu, rabbit monoclonal primary antibody; clone 4B5; Ventana) was performed.

The percentage and intensity of nuclear staining with ER and PR were estimated, and nuclear staining of ≥ 1% of the invasive tumor nuclei was interpreted as positive, in accordance with 2010 ASCO/CAP guidelines. HER2 positivity refers to cases with an IHC score of 3 + or fluorescence in situ hybridization (FISH) amplification (by 2013 ASCO/CAP criteria) for IHC scores of 2+. Patients were separated into three groups for the purposes of our analysis according to ER expression level: ER-negative, ER low positive (ER staining 1-10%), and ER > 10% positive (ER staining > 10%). The ER expression status on the surgical specimen was recorded. The assessments were performed locally by two experienced pathologists.

### Procedures

All patients received NAC after discussion at the weekly multidisciplinary team meeting according to the NCCN guidelines for invasive breast cancer. Most patients received 4 cycles of cyclophosphamide and anthracycline every 21 days, followed by 4 cycles of docetaxel every 21 days (AC-T) or 4 cycles of cyclophosphamide and anthracycline every 14 days, followed by 4 cycles of paclitaxel every 14 days (ddAC-P). Other chemotherapy regimens included 4 cycles of cyclophosphamide and anthracycline every 21 days and 6 cycles of paclitaxel and carboplatin. Subsequently, patients underwent appropriate breast and axillary surgery within 4 weeks of completion of NAC. All patients underwent either breast-conserving surgery (BCS) or mastectomy for the breast and underwent either sentinel lymph node biopsy (SLNB) or axillary lymph node dissection (ALND) for the axilla. Patients who underwent BCS or had histologically confirmed axillary disease received radiotherapy, and endocrine therapy after surgery was highly recommended to patients who had positive IHC for ER and/or PR.

### Pathologic complete response assessment and outcomes

Pathologic complete response (pCR) was defined as a complete absence of invasive tumor in the breast resection specimen and regional lymph nodes after neoadjuvant chemotherapy. Residual in situ carcinoma was allowed (ypT0/is, ypN0). The assessments were performed locally by two experienced pathologists. All patients were regularly followed up every three months for the first 2 years and then every 6 months. Follow-up was completed in March 2022. Both DFS and OS were included as clinical outcomes. DFS was defined as the time from surgery to invasive relapse (locoregional or distant), death, or last follow-up. OS was defined as the time from surgery to death or last follow-up.

### Statistical analysis

Statistical analysis was performed using SPSS version 24 (SPSS, Inc., Chicago, IL). Normally distributed data are described as the mean (standard deviation), and non-normally distributed data are described as the median (range). Continuous variables were compared using the Mann‒Whitney U test, and categorical data were compared using the chi-square test and Fisher’s exact test. Univariate and multivariate logistic regression analyses were used to assess the association between each variable and pCR. The variables with P values of ≤ 0.2 in the univariate analysis were included in the multivariable logistic regression analysis. Kaplan‒Meier survival curves for DFS and OS were analyzed with respect to ER levels, and P values were obtained using the log-rank test. Univariate and multivariate Cox proportional hazard models were used to determine the effects of prognostic factors on survival distributions. For all tests, a P value of less than 0.05 was considered statistically significant.

## Results

### Patients

A total of 241 patients were included in the analysis (Figure S1). Among these patients, 22 (9.1%) had tumors with low ER positivity, 159 (66.0%) had tumors with ER > 10% positivity, and 60 (24.9%) had tumors with ER negativity; 146 (60.6%) had stage II disease at diagnosis, and 95 (39.4%) had stage III disease. For the entire cohort, the median age at diagnosis was 51 years (range: 25–72). Most patients (215; 89.2%) received ACT (including AC-T and ddAC-P regimens) as NAC. Of the 22 patients with ER low positive tumors, 16 (72.7%) received adjuvant hormonal therapy, and 6 patients refused, including patients achieved pCR (4 patients) and patients with ER turned negative after NAC (2 patients), and all patients with ER > 10% positive tumors received adjuvant hormonal therapy.

The patients’ demographics and characteristics are shown in Table 1 with respect to ER expression level. Compared with patients with ER low positive tumors, patients with ER > 10% positive tumors had a lower Ki-67 labeling index (P = 0.028) and more PR-positive tumors (P < 0.001), and patients with ER-negative tumors had more advanced disease (P = 0.049) and more PR-negative tumors (P < 0.001).

The expression levels of ER in the residual tumors after NAC are shown in Table S1. one patient (1/159, 0.6%) turned ER-negative in initially ER > 10% positive patients. Three patients (3/60, 5.0%) turned ER-positive after NAC in ER-negative group. However, in ER low positive group, 5 (22.7%) patients turned ER-negative, and 3 (13.7%) patients turned ER > 10% positive.

**Table 1:** Characteristics of Patients with different Estrogen Receptor levels in Breast Cancer

Characteristic	ER low positive (n = 22)	ER > 10% positive (n = 159)	*P* Value ^a^	ER-negative (n = 60)	*P* Value ^b^
Age: Median (range), y	50.5 (29–66)	51 (25–72)	0.243	53 (25–72)	0.075
Ki-67 labeling index, Median (range), %	45 (3–80)	30 (1–90)	0.028*	60 (2–90)	0.402
Menopausal status					
Premenopausal	14	90	0.532	32	0.405
Postmenopausal	8	69		28	
Clinical stage at diagnosis					
II	16	101	0.397	29	0.049*
III	6	58		31	
Pretherapy primary tumor					
T1/T2	17	119	0.805	46	0.954
T3/T4	5	40		14	
Pretherapy lymph node status					
Negative ^c^	6	32	0.623	6	0.108
Positive	16	127		54	
PgR status					
Negative	9	14	< 0.001*	54	< 0.001*
Positive	13	145		6	
Neoadjuvant chemotherapy regimen					
ACT	20	143	1.000	52	0.889
Other	2	16		8	
Surgery					
Breast-conserving	7	48	0.876	15	0.537
Mastectomy	15	111		45	
Adjuvant hormonal therapy					
Yes	16	159	-	5	-
No	6	0		55	

ER, estrogen receptor; PgR, progesterone receptor; ACT, anthracycline, cyclophosphamide, taxane;

^a^ Comparisons between ER low positive and ER > 10% positive.

^b^ Comparisons between ER low positive and ER negative.

^c^ Including clinical negative and core biopsy negative.

### Pathologic complete response

A total of 35 patients (14.5%) achieved pCR after NAC. The pCR rates of ER low-positive tumors, ER > 10% positive tumors and ER-negative tumors were 31.8%, 6.3% and 30.0%, respectively. The pCR rate of ER low positive tumors was similar to that of ER-negative tumors (P = 0.874) and significantly higher than ER > 10% positive tumors (P = 0.001) (Table 2).

**Table 2:** Pathologic Complete Response (pCR) Rate in Different ER Expression Level

	No.	pCR, No. (%)	*P* Value (Comparison With low ER-positive)
ER low positive	22	7 (31.8)	Reference
ER > 10% positive	159	10 (6.3)	0.001*
ER negative	60	18 (30.0)	0.874

ER, estrogen receptor.

*Statistically significant.

In the univariate logistic regression analysis, ER > 10% positivity was significantly associated with low pCR rates compared with ER low positivity (OR, 0.144; 95% CI, 0.048–0.433; P = 0.001). A high Ki-67 labeling index as a continuous variable was significantly associated with high pCR rates (OR, 1.042; 95% CI, 1.025–1.059; P < 0.001). In addition, T1/T2 tumors and PR-negative tumors were also significantly associated with high pCR rates (Table 3).

The multivariate logistic regression model is also shown in Table 3. After adjustment for other covariates, ER low positive expression levels remained significantly associated with high pCR rates compared with ER > 10% positive (OR, 0.249; 95% CI, 0.067–0.923; P = 0.038), T1/T2 tumors, and negative lymph node status was also significantly associated with high pCR rates. There was no significant difference in pCR rates between the ER low positive and ER-negative groups in multivariate logistic regression (OR, 0.911; 95% CI, 0.230–3.609; P = 0.894).

**Table 3:** Predictors of pCR univariate and multivariate analysis

Variable	Univariable analysis		Multivariable analysis*	
	OR (95%CI)	*P* value	OR (95%CI)	*P* value
Age	1.004 (0.970–1.039)	0.826	-	-
Ki-67 labeling index	1.042 (1.025–1.059)	< 0.001**	1.032 (1.012–1.053)	0.002**
Menopausal status				
Premenopausal	1		-	-
Postmenopausal	0.967 (0.469–1.994)	0.927	-	-
Clinical stage at diagnosis				
II	1		-	-
III	0.773 (0.365–1.639)	0.502	-	-
Pretherapy primary tumor				
T1/T2	1		1	
T3/T4	0.158 (0.037–0.682)	0.013**	0.135 (0.028–0.645)	0.012**
Pretherapy lymph node status				
Negative	1		1	
Positive	0.477 (0.210–1.085)	0.078	0.314 (0.110–0.900)	0.031**
ER status				
ER low positive	1		1	
ER > 10% positive	0.144 (0.048–0.433)	0.001**	0.249 (0.067–0.923)	0.038**
ER-negative	0.918 (0.320–2.633)	0.874	0.911 (0.230–3.609)	0.894
PgR status				
Negative	1		1	
Positive	0.215 (0.101–0.457)	< 0.001**	0.727 (0.189-2.800)	0.643
Neoadjuvant chemotherapy regimen				
ACT	1		1	
Other	1.924 (0.713–5.192)	0.196	1.632 (0.494–5.393)	0.422

pCR, pathologic complete response; ER, estrogen receptor; PgR, progesterone receptor; ACT, anthracycline, cyclophosphamide, taxane; OR, odds ratio; CI, confidential interval;

*Multivariate analysis included variables with *P* < 0.20 in univariate analysis.

** Statistically significant.

### Survival analysis

**Fig. 1:**
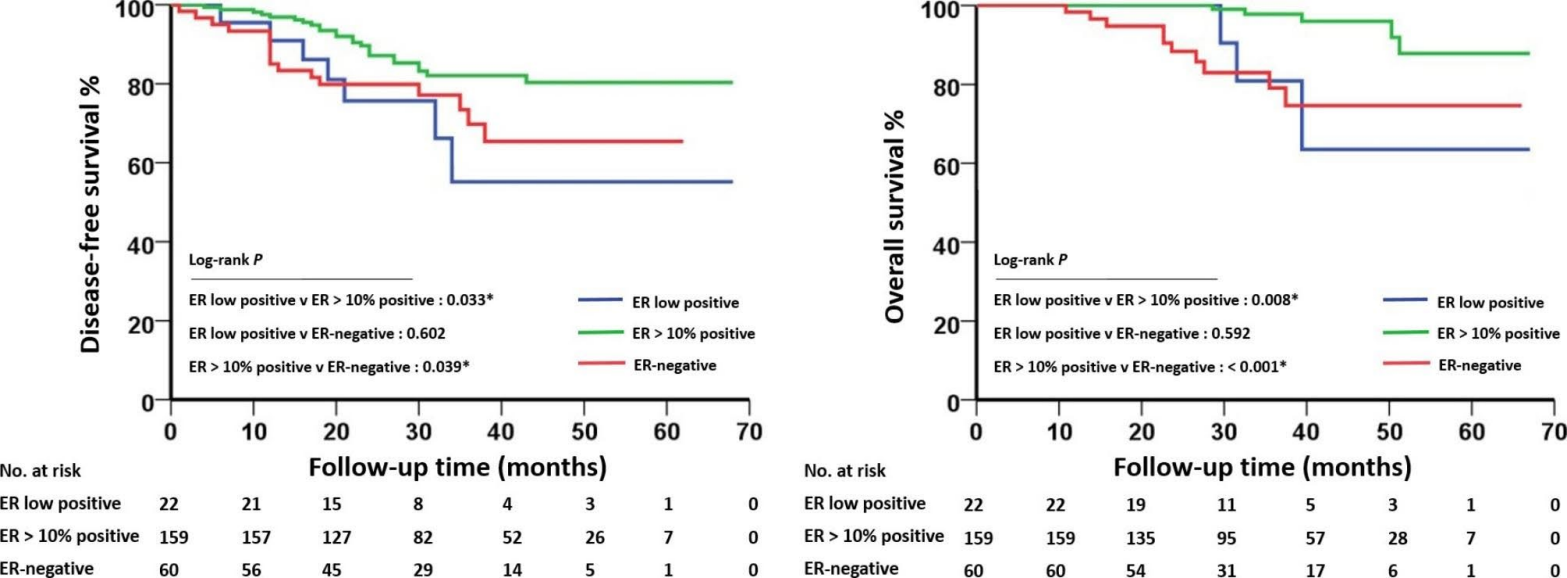
Disease-free survival (DFS) and overall survival (OS) analysis. (**A**) DFS and (**B**) OS curves for patients with ER low positive tumors, patients with ER > 10% positive tumors and patients with ER-negative tumors

The median follow-up time was 32 months. The DFS and OS of the patients with ER > 10% positive tumors were significantly better than those of the patients with ER-negative or ER low positive tumors. However, there were no significant differences between ER-negative or ER low positive tumors (Fig. 1).

The univariate Cox proportional hazard models revealed that compared with ER low positive tumors, ER > 10% positive tumors were significantly associated with better DFS (HR, 0.413; 95% CI, 0.178–0.959: P = 0.040) and OS (HR, 0.210; 95% CI, 0.050–0.887: P = 0.034), and patients with ER low positive and ER-negative tumors did not have significantly different DFS (HR, 0.791; 95% CI, 0.235–1.924: P = 0.605) or OS (HR, 1.532; 95% CI, 0.421–5.572: P = 0.517). Furthermore, PgR positivity was also associated with better OS than PgR negativity (Table S2). After adjustment for covariates, including age, Ki-67, stage, tumor size, lymph node status, ER status, PgR status and pCR status, ER > 10% positivity remained significantly associated with better DFS than ER low positivity (HR, 0.325; 95% CI, 0.131–0.807: P = 0.015) but was not significantly associated with OS (HR, 0.441; 95% CI, 0.070–2.764: P = 0.382). In addition, the multivariate Cox regression model revealed that pCR was significantly associated with better DFS (HR, 0.284; 95% CI, 0.081–0.993: P = 0.049) and OS (HR, 0.086; 95% CI, 0.009–0.790: P = 0.030) than non-pCR (Table 4).

**Table 4:** Multivariate Cox regression analysis of disease-free survival (DFS) and overall survival (OS)

Variable	DFS		OS	
	HR (95%CI)	*P* value	HR (95%CI)	*P* value
Age	0.970 (0.941–0.999)	0.040	0.961 (0.909–1.015)	0.153
Ki-67 labeling index	1.002 (0.988–1.017)	0.753	1.004 (0.980–1.028)	0.767
Clinical stage at diagnosis				
II	1			
III	1.606 (0.747–3.449)	0.225	1.422 (0.399–5.066)	0.587
Pretherapy primary tumor				
T1/T2	1			
T3/T4	0.738 (0.317–1.720)	0.481	0.382 (0.087–1.671)	0.201
Pretherapy lymph node status				
Negative	1		1	
Positive	2.899 (0.854–9.834)	0.088	3.193 (0.381–26.736)	0.284
ER status				
ER low positive	1		1	
ER > 10% positive	0.325 (0.131–0.807)	0.015*	0.441 (0.070–2.764)	0.382
ER-negative	0.822(0.286–2.362)	0.715	1.536 (0.324–7.286)	0.589
PgR status				
Negative	1		1	
Positive	1.212 (0.421–3.484)	0.722	0.266 (0.037–1.918)	0.189
Pathological response				
Non-pCR	1		1	
pCR	0.284 (0.081–0.993)	0.049*	0.086 (0.009–0.790)	0.030*

pCR, pathologic complete response; ER, estrogen receptor; PgR, progesterone receptor; HR, hazard ratio; CI, confidential interval;

* Statistically significant.

## Discussion

As an important prognostic and predictive biomarker in breast cancer, ER is evaluated in all cases of in situ and invasive diseases. It is recommended that laboratories should report both the percentage and intensity of hormone receptor staining in addition to the test interpretation as positive or negative. In previous studies, different definitions have been used to define the degree of ER positivity, except for the percentage of positively stained cells by IHC. A composite score such as the H score, Allred score, or quick score can also be provided to evaluate ER expression [6, 23–25]. However, composite scores have more influencing factors and lack a widely accepted definition of low ER expression. In our study, we used the percentage (1–10%) to define ER low positive status according to ASCO/CAP guidelines [15].

This study described clinicopathological characteristics, treatments, and outcomes of ER low positive tumors and compared them with ER > 10% positive and ER-negative disease. Previous studies have shown that ER low-expressing tumors present biological features and gene expression profiles similar to those of ER-negative tumors and are mostly classified as basal-like [16, 26, 27]. In our study, these ER low positive tumors presented a higher Ki-67 labeling index than ER > 10% positive tumors, but similar to ER-negative tumors, which showed that ER low positive tumors may have distinct characteristics that differ from the usual ER-positive cancers. However, pathological information such as grade and histological type were lacking in our study, and more data are needed to further confirm this point.

All patients included in our study were treated with neoadjuvant chemotherapy, and no difference was identified with respect to the type of chemotherapy regimens applied in the three subgroups. We noticed that ER expression status were changed in some patients after NAC. A lower percentage of conversion was observed in ER > 10% positive and ER-negative groups (1/159, 0.6% and 3/60, 5.0%, respectively). However, in ER low positive group, 5 (22.7%) patients turned ER-negative, and 3 (13.7%) patients turned into ER > 10% positive. Although many previous studies have reported the discordance of the ER status between before and after NAC, especially the conversion from ER-positive to ER-negative [28–30]. Our study further suggests that patients with low ER expression may have a higher probability of conversion. It indicates that this type of tumor may have stronger heterogeneity, further research on this conversion would be valuable.

It is well known that ER-positive/HER2-negative breast cancers present lower pCR rates than triple-negative breast cancers when treated with neoadjuvant chemotherapy [31], and a retrospective study showed that a low percentage of ER expression is associated with a high pCR rate. Our study indicated that the ER low positive tumors receiving neoadjuvant chemotherapy showed a pCR rate of 31.8%, which is consistent with previous studies that have described the response to neoadjuvant chemotherapy in ER low positive breast cancers [23, 27, 32, 33]. This value of 31.8% was higher than that for ER > 10% positive tumors (6.3%) but similar to the value for ER-negative tumors (30.0%). Meanwhile, previous studies reported pCR rates ranging between 26 and 41% in ER-negative breast cancer [32, 33] and 4-7% in classical ER-positive breast cancer [23, 32, 33], in line with our results. However, multivariate analysis showed that ER low positive expression levels were significantly associated with high pCR rates compared with ER > 10% positive expression, indicating that ER low positive breast cancer has a better response to NAC compared with classical ER-positive breast cancer. These results suggested that ER low positive tumors should be treated differently from classical ER-positive tumors.

Many studies have demonstrated that ER-positive tumors have a better prognosis than ER-negative tumors. However, because of the low frequency of ER low positivity, the prognosis for these patients is ambiguous. Our study demonstrated that patients with ER low positive tumors had significantly worse DFS and OS than those with ER > 10% positive tumors, but similar outcomes as the ER-negative group. These results are in accord with several previous studies that focused on patients with low ER expression [12, 18, 23, 33]. These data suggest that breast cancers with low ER positivity and those with ER negativity have similar molecular features and clinical prognoses, and the threshold of 1% stained cancer cells to define ER-positive status cannot accurately reflect the biological and clinical characteristics of tumors.

The ASCO/CAP guidelines have determined the threshold for positivity of ER/PR assays by IHC to be 1%. However, the clinical significance of the substantial benefit of endocrine therapy in ER low positive cases is equivocal. Dowsett et al. found that low ER or PgR expression is associated with a high risk of recurrence with either anastrozole or tamoxifen treatment [6]. Bouchard-Fortier et al. reported that breast cancer patients with weak ER expression (< 10 fmol/mg cytosol protein) do not significantly benefit from adjuvant antihormonal therapy (tamoxifen) compared with those exhibiting higher ER levels (≥ 10 fmol/mg cytosol protein) on a ligand-based assay (LBA) [34]. Chen et al. indicated that primary breast cancer patients with borderline ER-positive (1–9%) expression gained no significant survival benefit from endocrine therapy [35]. In addition, Cai et al. suggested that short-term endocrine therapy for 2 to 3 years might be an alternative for patients who have ER low positive breast cancer instead of the standard 5 years of treatment [36]. In our study, all patients with ER > 10% positive tumors received endocrine therapy, but only 72.7% (16/22) of ER low positive patients and 8.3% (5/60) of ER-negative patients received endocrine therapy. Unfortunately, the number of ER low positive patients was too small to detect statistically significant differences in DFS or OS between patients who received adjuvant endocrine therapy and those who did not. However, in view of its side effects, we must carefully assess the benefit in ER low positive patients and make an appropriate decision about the administration of endocrine therapy. Further study is needed to elucidate the ideal application of endocrine therapy in ER low positive patients.

In addition to ER status, PgR status is also an important marker for breast cancer. PgR is a ligand-activated nuclear transcription factor expressed in over two-thirds of ER-positive breast cancers [37, 38]. It is known as an estrogen-regulated gene, and the dependence of PgR expression on ER activity means that ER and PgR expression are typically concordant [39]. However, 20% of invasive breast cancers exhibit discordant hormone receptor statuses, and most are ER-positive/PgR-negative subgroups. In this study, PgR was expressed in 59.1% of ER low positive tumors, which was significantly higher than that in ER-negative tumors but lower than that in ER > 10% positive tumors, indicating that PgR is related to the degree of ER expression. Univariable analysis showed that PgR positivity was associated with a lower pCR rate, and univariate Cox regression analysis indicated that PgR positivity was associated with better OS, but the results of multivariate analysis did not support this, which may be due to the strong correlation between ER and PgR.

The current study has several limitations. First, it was a retrospective study, and treatment was not randomly assigned. Because of the low frequency of ER low positive breast cancer, conducting a randomized controlled trial that targets this cohort would be extremely difficult. We look forward to this type of study. Second, the sample size of ER low positive patients was too small, which may make it difficult to detect statistically significant differences. Third, data including the pathological information of tumors, such as tumor grade and histological type, were missing in our study, and we could not well demonstrate the difference in pathological features in tumors with different ER expression. Last, the definition for low expression of ER is controversial: some previous studies use 1–9%, and others use 1–10%. The ASCO/CAP classifies ER low positive status as 1–10% of cells with positive staining. However, the current recommendations for defining ER positivity are based on retrospective analyses, and the threshold is still equivocal.

In summary, our results showed that ER low positive breast cancer presents a substantially better response to NAC than classical ER-positive (ER > 10% positive) breast cancer, and patients with ER low positive have a significantly worse prognosis than those with ER > 10% positive tumors, but a prognosis similar to that of the ER-negative group. These data support that this category of patients should be treated differently from those with ER > 10% positive tumors, and further prospective study is anticipated.

### Electronic supplementary material

Below is the link to the electronic supplementary material.

Supplementary Material 1

Supplementary Material 2

Supplementary Material 3

## Data Availability

The data that support the findings of this study are available from the corresponding authors upon reasonable request.
