# Supplementary figures and images for: Impact of estrogen receptor expression level on response to neoadjuvant chemotherapy and prognosis in HER2-negative breast cancers
Source: BMC Cancer. 2023 Sep 8;23:841. doi: 10.1186/s12885-023-11368-2 (PMC10485958; doi:10.1186/s12885-023-11368-2)

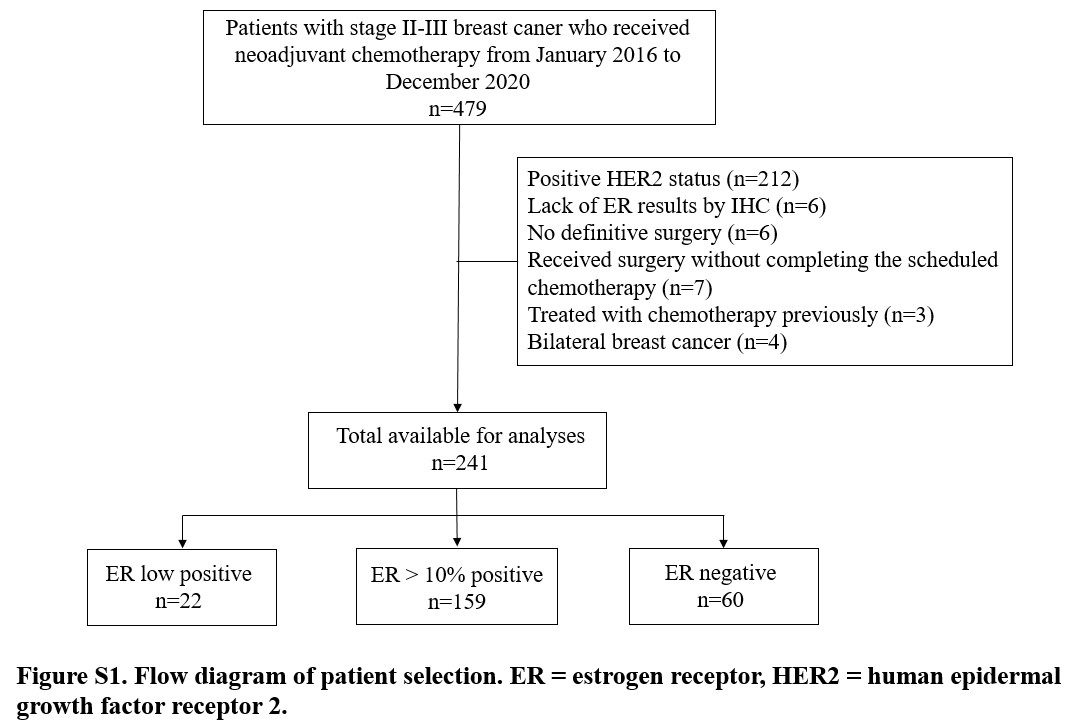

Supplement: Supplementary file 1 — Supplementary Material 1 [file 12885_2023_11368_MOESM1_ESM.jpg]
