# Supplementary material for: Impact of estrogen receptor expression level on response to neoadjuvant chemotherapy and prognosis in HER2-negative breast cancers
Source: BMC Cancer. 2023 Sep 8;23:841. doi: 10.1186/s12885-023-11368-2 (PMC10485958; doi:10.1186/s12885-023-11368-2)
Supplement: Supplementary file 2 — Supplementary Material 2 [file 12885_2023_11368_MOESM2_ESM.docx]

**Supplementary Table S1. The ER expression level in the residual tumors after neoadjuvant chemotherapy (NAC)**

|  | No. | pCR, No. (%) | The ER expression level after NAC, No. (%) | | |
| --- | --- | --- | --- | --- | --- |
|  |  |  | ER low positive | ER > 10% positive | ER negative |
| ER low positive | 22 | 7 (31.8) | 7 (31.8) | 3 (13.7) | 5 (22.7) |
| ER > 10% positive | 159 | 10 (6.3) | 144 (90.6) | 4 (2.5) | 1 (0.6) |
| ER negative | 60 | 18 (30.0) | 1 (1.7) | 2 (3.3) | 39 (65.0) |

ER, Estrogen Receptor
