# Supplementary material for: Impact of estrogen receptor expression level on response to neoadjuvant chemotherapy and prognosis in HER2-negative breast cancers
Source: BMC Cancer. 2023 Sep 8;23:841. doi: 10.1186/s12885-023-11368-2 (PMC10485958; doi:10.1186/s12885-023-11368-2)
Supplement: Supplementary file 3 — Supplementary Material 3 [file 12885_2023_11368_MOESM3_ESM.docx]

**Supplementary Table S2. Univariate Cox regression analysis of disease-free survival (DFS) and overall survival (OS)**

| **Variable** | **DFS** | | **OS** | |
| --- | --- | --- | --- | --- |
|  | **HR (95%CI)** | ***P* value** | **HR (95%CI)** | ***P* value** |
| Age | 0.975 (0.947-1.002) | 0.073 | 0.978 (0.934-1.025) | 0.355 |
| Ki-67 labeling index | 1.005 (0.993-1.017) | 0.400 | 1.024 (1.005-1.043) | 0.012* |
| Menopausal status |  |  |  |  |
| Premenopausal | 1 |  | 1 |  |
| Postmenopausal | 0.790 (0.453-1.432) | 0.437 | 0.746 (0.282-1.970) | 0.554 |
| Clinical stage at diagnosis |  |  |  |  |
| II | 1 |  |  |  |
| III | 1.665 (0.939-2.951) | 0.081 | 1.454 (0.577-3.665) | 0.428 |
| Pretherapy primary tumor |  |  |  |  |
| T1/T2 | 1 |  |  |  |
| T3/T4 | 1.334 (0.703-2.530) | 0.377 | 1.019 (0.335-3.102) | 0.974 |
| Pretherapy lymph node status |  |  |  |  |
| Negative | 1 |  | 1 |  |
| Positive | 3.583 (1.112-11.546) | 0.033* | 4.968 (0.661-37.365) | 0.119 |
| ER status |  |  |  |  |
| ER low positive | 1 |  | 1 |  |
| ER > 10% positive | 0.413 (0.178-0.959) | 0.040* | 0.210 (0.050-0.887) | 0.034* |
| ER-negative | 0.791 (0.235-1.924) | 0.605 | 1.532 (0.421-5.572) | 0.517 |
| PgR status |  |  |  |  |
| Negative | 1 |  | 1 |  |
| Positive | 0.644 (0.357-1.161) | 0.143 | 0.146 (0.055-0.388) | < 0.001* |
| Neoadjuvant chemotherapy regimen |  |  |  |  |
| ACT | 1 |  | 1 |  |
| Other | 0.908 (0.359-2.298) | 0.838 | 0.366 (0.049-2.751) | 0.329 |
| Surgery |  |  |  |  |
| Mastectomy | 1 |  | 1 |  |
| Breast-conserving | 0.824 (0.427-1.588) | 0.562 | 0.812 (0.291-2.267) | 0.691 |
| Pathological response |  |  |  |  |
| Non-pCR | 1 |  | 1 |  |
| pCR | 0.403 (0.125-1.298) | 0.128 | 0.353 (0.047-2.645) | 0.311 |

pCR, Pathologic Complete Response; ER, Estrogen Receptor; PgR, Progesterone receptor; HR, hazard ratio; CI, confidential interval;

* Statistically significant.
